# Risk factors for vaginal fistula symptoms in Sub-Saharan Africa: a pooled analysis of national household survey data

**DOI:** 10.1186/s12884-016-0871-6

**Published:** 2016-04-21

**Authors:** Mathieu Maheu-Giroux, Véronique Filippi, Nathalie Maulet, Sékou Samadoulougou, Marcia C. Castro, Nicolas Meda, Mariève Pouliot, Fati Kirakoya-Samadoulougou

**Affiliations:** Department of Infectious Disease Epidemiology, Imperial College London, St Mary’s Hospital, London, UK; Department of Infectious Disease Epidemiology, London School of Hygiene and Tropical Medicine, London, UK; Institute of Health and Society, Université Catholique de Louvain, Clos Chapelle-aux-champs, Brussels, Belgium; Pôle Épidemiologie et Biostatistique, Institute de recherche expérimentale et Clinique, Université Catholique de Louvain, Clos Chapelle-aux-champs, Brussels, Belgium; Department of Global Health and Population, Harvard TH Chan School of Public Health, Boston, MA USA; Centre Muraz, Ministry of Health, Bobo-Dioulasso, Burkina Faso; UFR Sciences de la Santé, Université de Ouagadougou, Ouagadougou, Burkina Faso; Institute of Food and Resources Economics, Section for Global Development, University of Copenhagen, Copenhagen, Denmark; Faculté de Santé Publique, Université catholique de Louvain, Brussels, Belgium

**Keywords:** Obstetric fistula, vesicovaginal fistula, rectovaginal fistula, reproductive health, sexual health, women’s health

## Abstract

**Background:**

Vaginal fistula (VF) is one of the most severe maternal morbidities with the immediate consequence of chronic urinary and/or fecal incontinence. The epidemiological evidence regarding risk factors for VF is dominated by facility-based studies. Our aim is to estimate the effect size of selected risk factors for VF using population-based survey data.

**Methods:**

We pooled all available Demographic and Health Surveys and Multiple Indicators Cluster Surveys carried out in sub-Saharan Africa that collected information on VF symptoms. Bayesian matched logistic regression models that accounted for the imperfect sensitivity and specificity of self-reports of VF symptoms were used for effect size estimation.

**Results:**

Up to 27 surveys were pooled, including responses from 332,889 women. Being able to read decreased the odds of VF by 13 % (95 % Credible Intervals (CrI): 1 % to 23 %), while higher odds of VF symptoms were observed for women of short stature (<150 cm) (Odds Ratio (OR) = 1.31; 95 % CrI: 1.02-1.68), those that had experienced intimate partner sexual violence (OR = 2.13; 95 % CrI: 1.60-2.86), those that reported sexual debut before the age of 14 (OR = 1.41; 95 % CrI: 1.16-1.71), and those that reported a first birth before the age of 14 (OR = 1.39; 95 % CrI: 1.04-1.82). The effect of post-primary education, female genital mutilation, and having problems obtaining permission to seek health care were not statistically significant.

**Conclusions:**

Increasing literacy, delaying age at first sex/birth, and preventing sexual violence could contribute to the elimination of obstetric fistula. Concomitant improvements in access to quality sexual and reproductive healthcare are, however, required to end fistula in sub-Saharan Africa.

**Electronic supplementary material:**

The online version of this article (doi:10.1186/s12884-016-0871-6) contains supplementary material, which is available to authorized users.

## Background

In sub-Saharan Africa, maternal disorders are the second most important cause of death among women of reproductive age (15-49 years old) [1]. Disease burden attributable to maternal complications still remains important despite the significant declines in maternal mortality observed in this region since the mid-2000s [2, 3]. In fact, it is estimated that for every woman dying from maternal complications, another 20 women will have to withstand serious maternal morbidity [4]. Of all maternal morbidities, obstetric fistula is one of the most debilitating conditions with the immediate consequence of chronic urinary and/or fecal incontinence. Physical comorbidities, psychological distress, and social stigmatization usually follow [5–9].

The etiology of vaginal fistula (VF), an abnormal hole between the bladder (vesico-vaginal fistula) and/or rectum (recto-vaginal fistula) and the reproductive tract of a woman, is divided into two main categories: obstetric and traumatic. VF of obstetric origin are caused by an intertwined set of biological, socio-economic, and cultural factors that favor obstructed labor and triggered by insufficient or delayed access to quality emergency obstetric care [7]. VF of traumatic origin mostly results from sexual violence. The vast majority of VF in sub-Saharan Africa are of obstetric origins and prevalence of this condition in this region was recently estimated to be between 1.0 and 1.6 per 1,000 women of reproductive age depending on methodology [10, 11].

The epidemiological evidence regarding risk factors for VF is dominated by facility-based studies [12]. The numerous clinical series usually report socio-demographic characteristics of VF patients (age of marriage, marital status, literacy, parity, etc.) as well as circumstances of fistula occurrence (duration of labor, type of birth attendance, mode and place of delivery, etc.) [13–21]. This accumulation of hospital-based studies contributed to highlight the diverse characteristics of fistula sufferers who present to facilities [12]. A few case-control studies tackle individual determinants with the aim to confirm risk factors [22–24] or develop a fistula prevention index [25]. Other studies, often qualitative, reflect on cultural or health system factors to reduce the three delays causative of obstetric fistula and maternal mortality [21, 26–28]: delay in decision to seek care, delay in reaching care, and delay in receiving adequate care once in the health facility.

Population-based studies could be less susceptible to selection bias than case series from facility and case-control studies but are rarely carried-out [11, 29]. In sub-Saharan Africa, the main sources of nationally representative health data are Demographic and Health Surveys (DHS) and Multiple Indicator Cluster Surveys (MICS). Since 2004, these surveys progressively began to include questions about VF symptoms. These data sources were recently used to estimate the prevalence of VF in sub-Saharan Africa [10], while adjusting for the imperfect accuracy of self-reports, but a thorough and systematic examination of individual risk factors has yet to be completed. We are aware of four population-based studies that examined determinants of VF [30–33]. These studies only included a small number of surveys, assumed that self-reports of VF symptoms were perfectly accurate, and none pooled surveys together, severely limiting their statistical power.

The primary objective of this paper is thus to examine the association between selected individual risk factors and lifetime prevalence of self-reported VF symptoms, such as literacy status, education level, female genital mutilation (FGM), sexual violence, short stature, age at first sexual intercourse, age at first birth, and women’s ability to get permission to seek health care. By pooling surveys from different countries, we hope to improve the representativeness and precision of the effect size measures for those risk factors.

## Methods

### Data sources

DHS and MICS surveys conducted in sub-Saharan Africa that included questions about VF symptoms were considered for this analysis. A comprehensive overview of DHS and MICS surveys can be found elsewhere [34]. Briefly, both DHS and MICS are household-based surveys that use a multistage stratified cluster sampling design to select a nationally representative sample of women of reproductive age (15-49 years old). Socio-demographic characteristics and information on selected health indicators are collected through face-to-face interviews by trained personnel and recorded in standard questionnaires. The majority of surveys administered the VF questions to all women of reproductive age but some restricted it to women that were ever married (Mauritania MICS 2011), ever pregnant (Swaziland MICS 2010 and Guinea-Bissau MICS 2010), or that had a live birth in the previous five years (Rwanda DHS 2005).

### Procedures

The specific questions related to vaginal fistula symptoms varied slightly from survey to survey and a contingency question about knowledge of vaginal fistula was sometime incorporated. A full description of the VF and contingency questions (if any), their probes, and the coding of the outcome can be found elsewhere [10].

Based on previous studies and the information available from DHS/MICS surveys, we estimated the effect of the following risk factors: illiteracy, education level, whether the respondent has experienced female genital mutilation (FGM), short stature, experience of intimate partner sexual violence, young age at first sexual intercourse, young age at first birth, and women’s difficulty to get permission to access health care. Literacy status was ascertained in the surveys by asking the interviewee to read a sentence on a card that was handed out to her. If the woman was able to read only part of the sentence, she was considered not being able to read properly. Women who reported having had some secondary education or higher were de facto assumed to be literate. For genital mutilation, we did not stratify our analysis by FGM type as a validation study of the DHS FGM questions in Sierra Leone demonstrated that they were accurate to determine FGM prevalence but inaccurate for determining cutting extent [35]. Not all surveys recorded information for these risk factors and the list of countries for which such data was collected is presented in Tables 1 and 2. As for women’s anthropometric measurements, this information is not collected by MICS surveys and the women’s height was recorded from a sub-sample of participants in most DHS surveys. Similarly, questions on domestic violence were often administered to a subsample of women, depending on the survey, and the questions about ever having experienced intimate partner sexual violence were only asked to ever married women (or those in a union). As for age at first sexual intercourse, inconsistent responses were disregarded and considered as missing (e.g., a women reporting never having had sexual intercourse but having given birth). Finally, most DHS surveys asked women if getting permission to seek health care was a problem. Those who responded that it was a big problem were considered as having limited ability to seek the care they need.

**Table 1:** Number of vaginal fistula (VF) by survey datasets for the following risk factors: literacy status, education level, female genital mutilation (FGM), and short stature (<150 cm)

Country, Survey, and Year	Literate		Post-primary education		FGM		Short Stature	
	VF (N)/Literate (N)	VF (N)/Illiterate (N)	VF (N)/Post-primary (N)	VF (N)/No Post-primary (N)	VF (N)/FGM (N)	VF (N)/No FGM (N)	VF (N)/Short (N)	VF (N)/Tall (N)
Benin DHS 2011-12	36/4182	91/12417	31/3450	96/13149	10/1370	117/15229	12/1115	111/15061
Burkina Faso DHS 2010	6/3063	14/13982	6/2201	14/14854	17/12884	3/4176	0/243	10/8202
Cameroon DHS 2011	32/9212	24/6163	27/7148	29/8271	Not Measured		1/411	25/7473
Chad MICS 2010	6/2334	34/13437	5/1857	35/14000	24/7218	16/8569	Not Measured	
Comoros DHS 2012	50/2974	59/2319	49/2761	60/2547	Not Measured		16/590	92/4593
Congo (Brazz.) DHS 2011-12	17/6740	10/4052	14/6299	13/4519	Not Measured		2/513	12/5134
DRC DHS 2007	22/5015	22/4954	19/4107	25/5879	Not Measured		3/782	12/3949
Ethiopia DHS 2005	21/3937	82/10103	10/2650	93/11405	69/10012	32/3603	1/852	44/5798
Guinea DHS 2012	10/1812	52/7306	10/1703	53/7432	63/8935	0/194	4/256	27/4457
Kenya DHS 2008-09	54/5809	24/2609	21/2796	57/5640	19/2539	59/5891	5/569	72/7742
Malawi DHS 2010	59/13471	73/9500	14/4289	118/18721	Not Measured		7/1136	28/6496
Mali DHS 2006	0/1809	18/12730	0/1546	18/13034	18/11740	0/2827	17/13808	17/13808
Mali DHS 2012-13	12/1810	59/8614	9/1689	62/8735	69/9480	2/944	0/138	28/5134
Mauritania DHS	4/3459	3/5537	2/1539	5/7557	4/6702	3/2377	Not Measured	
Niger DHS 2006	2/1185	18/7985	1/967	19/8222	2/209	18/8952	0/179	12/4351
Niger DHS 2012	0/1672	16/9432	0/1373	16/9759	1/209	15/10924	0/214	7/4912
Nigeria DHS 2008	55/14345	86/18804	51/13527	91/19790	37/8452	102/24709	20/3853	120/28572
Rwanda DHS 2005	80/3041	84/2332	12/519	152/4867	Not Measured		6/273	66/2422
Senegal DHS 2010-11	3/3983	15/11705	1/2802	17/12886	11/5689	7/9999	0/126	8/5633
Sierra Leone DHS 2013	23/5415	89/11085	22/5206	90/11337	105/14773	6/1760	4/764	46/7185
Swaziland MICS 2010	48/2905	9/395	36/2195	21/1123	Not Measured		Not Measured	
Tanzania DHS 2010	32/6903	18/3205	7/2360	44/7776	9/1322	42/8807	13/1636	37/8408
Togo MICS 2010	4/2255	19/4108	4/1744	19/4631	0/393	23/5970	Not Measured	
Togo DHS 2013-2014	35/3579	58/5874	27/3070	66/6404	6/602	87/8861	2/314	50/4517
Uganda DHS 2006	80/3867	121/4606	27/1823	174/6653	2/61	199/8403	11/248	63/2596
Uganda DHS 2011	76/4298	88/4307	32/2509	132/6097	7/156	156/8423	8/194	51/2493
Zambia DHS 2013-2014	41/9554	49/6774	34/7386	57/9001	Not Measured		12/1677	79/14562

VF = Vaginal Fistula; FGM = Female genital mutilation; DHS = Demographic and Health Survey; MICS = Multiple Indicators Cluster Survey

The survey-specific total sample sizes can vary by risk factor depending on the number of missing observations and eligibility criteria

**Table 2:** Number of vaginal fistula (VF) by survey datasets for the following risk factors: experience of intimate partner sexual violence (IPSV), young age at first sex (<14 years old), young age at first birth (<14 years old), and permission to seek health care

	IPSV^a^		Young age at 1^st^ intercourse^b^		Young age at 1^st^ birth^c^		Permission to seek health care	
Country, Survey, and Year	VF (N)/Sex Violence (N)	VF (N)/No Violence (N)	VF (N)/Young (N)	VF (N)/Old (N)	VF (N)/Young (N)	VF (N)/Old (N)	VF (N)/Big problem (N)	VF (N)/Not big problem (N)
Benin DHS 2011-12	Not Measured		17/1250	83/11681	5/604	84/11918	55/5797	72/10802
Burkina Faso DHS 2010	0/142	11/9859	0/523	18/13971	0/106	16/13129	7/3850	13/13208
Cameroon DHS 2011	5/574	9/3425	3/1396	49/11148	2/526	47/10492	7/2251	22/5195
Chad MICS 2010	1/844	33/11283	5/2165	33/11729	5/908	31/11933	Not Measured	
Comoros DHS 2012	2/36	61/2492	9/243	75/3128	2/110	75/2818	34/2405	73/2885
Congo (Brazz.) DHS 2011-12	Not Measured		5/1510	22/8405	0/250	24/8537	15/4752	12/6056
DRC DHS 2007	7/764	10/2082	9/958	32/7268	3/250	32/6890	10/2115	34/7861
Ethiopia DHS 2005	Not Measured		22/1892	72/8172	6/362	87/8964	38/3999	65/10049
Guinea DHS 2012	Not Measured		18/1415	41/6383	5/368	52/6576	Not Measured	
Kenya DHS 2008-09	18/626	39/4273	10/597	61/5890	2/189	73/5906	Not Measured	
Malawi DHS 2010	7/842	19/4531	22/1904	107/16772	5/536	121/17496	22/2571	110/20421
Mali DHS 2006	0/307	13/8613	0/1554	17/10609	0/525	17/11039	0/2764	18/11787
Mali DHS 2012-13	1/378	19/2742	7/1108	56/7151	3/480	64/8000	30/3063	41/7361
Mauritania MICS 2011	Not Measured		Not Measured		Not Measured		Not Measured	
Niger DHS 2006	Not Measured		2/1294	15/6225	1/256	16/6929	1/860	19/8315
Niger DHS 2012	Not Measured		5/1160	10/8356	5/338	11/8865	4/2429	12/8713
Nigeria DHS 2008	5/688	88/18509	21/3221	102/23255	5/1144	114/22552	15/4775	127/28412
Rwanda DHS 2005	14/257	33/1822	0/88	156/5134	0/17	164/5369	9/102	154/5278
Senegal DHS 2010-11	Not Measured		4/1505	12/9717	1/600	16/10052	7/2576	11/13112
Sierra Leone DHS 2013	3/248	39/4027	11/1358	87/12246	9/697	94/11570	30/2686	82/13831
Swaziland MICS 2010	Not Measured		5/118	52/3198	0/9	57/3270	Not Measured	
Tanzania DHS 2010	7/695	25/4991	2/498	40/7639	1/96	42/7227	1/229	50/9899
Togo MICS 2010	Not Measured		1/299	22/5417	2/124	18/4592	Not Measured	
Togo DHS 2013-14	12/420	52/4949	6/490	76/7416	2/151	78/6789	10/1176	83/8294
Uganda DHS 2006	17/516	25/1225	32/769	158/6214	6/173	189/6231	24/653	177/7815
Uganda DHS 2011	15/423	21/1271	22/822	127/5997	8/292	137/6076	12/475	152/8127
Zambia DHS 2013-14	18/1502	53/7896	4/894	76/12205	3/191	78/12220	4/491	87/15882

VF = Vaginal Fistula; IPSV = Intimate Partner Sexual Violence; DHS = Demographic and Health Survey; MICS = Multiple Indicators Cluster Survey

^a^Among married and/or ever married women (or those in a union)

^b^Among sexually active women

^c^Among primi/multiparous women

The survey-specific total sample sizes can vary by risk factor depending on the number of missing observations and eligibility criteria

The principal threat to the internal validity of our analyses is confounding of the exposure-outcome relationship. The main potential confounders for which information was collected by the survey questionnaires are age, literacy status, location of residence (rural versus urban), gravidity status, and religion. Socio-economic status and marital status were not considered in this analysis because these variables are likely both a cause and an effect of VF. That is, due to the cross-sectional nature of data collection, we do not have information on the temporal sequence in which changes in socio-economic status or marital status occurred. Three surveys (Chad MICS 2010, Mauritania MICS 2011, and Togo MICS 2010) did not record information on gravidity status and we assumed that all nulliparous women were also nulligravid – a reasonable assumption giving the high correlation observed between these two variables. Finally, four surveys did not record information on religion and these were coded using a missing variable indicator to retain them in the analyses (Mauritania MICS 2011, Niger DHS 2012, Swaziland MICS 2010, and Tanzania DHS 2010).

### Statistical analyses

To circumvent the lack of balance and overlap for some of the covariates, matching was used to make the group with the selected risk factor (i.e., exposed) as similar as possible to the group without (i.e., unexposed). By reducing model dependency through this semi-parametric data preprocessing, we aim to produce more robust inferences that are less sensitive to modeling assumptions [36]. Three of our risk factors are continuous and were dichotomized. Respondents with a height less than 150 cm, a commonly used threshold [12, 15], were defined as having a short stature. For age at first birth, visual inspection of the exposure-outcome relationship suggested that this variable could be dichotomized at less than 14 years of age at first delivery. This corresponds roughly to the 4^th^ percentile of the distribution of age at first birth. The same threshold of less than 14 years was used to define young age at first sexual intercourse.

All country datasets were pooled together as the low number of VF cases precludes data analysis at the country level for many surveys (i.e., all cases were either exposed or unexposed in these surveys). For the selected risk factors, a nearest neighbor algorithm was used to match women on sampling weight (for sexual violence, the sampling weight from the domestic violence questionnaire was used), age (continuous), and survey identifier. For this latter variable, exact matching was used for risk factors that consistently had more unexposed than exposed observations across surveys: short stature, intimate partner sexual violence, young age at first sexual intercourse, young age at first birth, and problem obtaining permission to seek care (otherwise, nearest neighbor matching was used). The matching ratio of exposed to unexposed units varied for each risk factor and was chosen as to minimize unbalance and maximize statistical power. Matching was implemented using the ‘*MatchIt*’ package [37] in R. Unmatched women were excluded from the analyses.

Logistic regression models were used on the matched data to estimate the effect of the selected risk factors on lifetime prevalence of VF. Missing values for the selected risk factors and covariates were always less than 1 %, except for height (2.0 %) and age at first sexual intercourse (6.1 % of inconsistent or missing values). Observations with missing values were excluded from the analyses (with the exception of those for religion which were retained using a missing indicator). To provide for additional control of potential confounders, we adjusted for the following covariates: age (15-19, 20-29, and 30-49 years), literacy status (this covariate was not included for literacy status and education level risk factors), gravidity status (not included for age at first birth), location of residence (urban/rural), religion (Christian, Muslim, others, missing), and the survey’s country. Such analyses have been described as doubly-robust because statistically consistent inferences can be made “*if either the matching analysis or the analysis model is correct (but not necessarily both)*” [37]. Surveys that had a different population denominator were included in the analysis since we matched on survey identifier and country fixed effects were included in the parametric analyses. These logistic regressions did not account for the clustered design of surveys as our preliminary analyses have shown that clustering the standard errors had no impact on our conclusions (also discussed in [10]).

Importantly, women’s self-report of vaginal fistula symptoms do not have perfect sensitivity and specificity, as compared to the gold standard of a pelvic examination. In order to account for non-differential misclassification of the self-reported outcome, we used a latent-class Bayesian statistical model [10, 38, 39]. The underlying assumption being that all surveys have a common sensitivity and specificity (see [10] for details). This model takes the following form:

$$ \begin{array}{c}\mathrm{Likelihood}:\\ {}{y}_i\sim Binomial\left({p}_i,{N}_i\right)\\ {}{p}_i={\pi}_i(Se)+\left(1-{\pi}_i\right)\left(1-Sp\right)\\ {}\mathrm{logit}\left({\pi}_i\right)=\alpha +\beta {X}_i\end{array} $$

Because of our very large sample sizes and the computing-intensive nature of Bayesian calculations, we grouped observations with the same covariate patterns and used a binomial likelihood instead of the standard Bernoulli (i.e., grouped logistic regression). In this model, *y*_*i*_ is the total number of women reporting VF symptoms with covariate pattern *i*; *N*_*i*_ is the total number of women with covariate pattern *i*; *p*_*i*_ is the observed probability of reporting VF symptoms, *π*_*i*_ is the true probability of women having ever had VF symptoms; *Se* and *Sp* are the sensitivity and specificity of the survey instrument, respectively; *α* is the model’s intercept; *β* is a vector of coefficients for the covariates included in *X*_*i*_. The model’s specification is completed using the following prior distributions:

$$ \begin{array}{c}\mathrm{Prior}\ \mathrm{distributions}\ \mathrm{f}\mathrm{o}\mathrm{r}\ \mathrm{model}\ \mathrm{parameters}:\\ {}\alpha \sim Normal\left(0,20\right)\\ {}\beta \sim Normal\left(0,20\right)\\ {}Se\sim Uniform\left(95.10\%,99.90\%\right)\\ {}Sp\sim Uniform\left(99.85\%,99.95\%\right)\end{array} $$

Both *α* and *β* are given non-informative priors that follow a normal distribution with a mean of zero and standard deviation of 20. For sensitivity and specificity, we used uniform distributions that match the 95 % credible intervals of the posterior distributions of these quantities, as estimated previously [10]. Posterior distributions were obtained using Markov Chain Monte Carlo sampling, implemented in R using the ‘rstan’ package [40]. Samples are obtained using the no-U-turn sampler, a computationally efficient variant of Hamiltonian Monte Carlo [41]. Inferences were based on three chains of 30,000 samples after an initial warm-up period of 2,500 samples per chain (total of 90,000 iterations used for inferences). Convergence was examined using traceplots and ensuring that the potential scale reduction factor was equal to one. All analyses were performed using the R statistical software [42].

## Results

### Surveys characteristics

A total of 31 surveys collected information on VF symptoms in sub-Saharan Africa. Of these, individual data records were not available for two surveys (Equatorial Guinea DHS 2011 and Guinea-Bissau MICS 2010), and two other surveys were excluded because the incontinence questions were considered to be non-specific (Côte d’Ivoire DHS 2011-12 and Malawi DHS 2004). Hence, 27 surveys, conducted between 2005 and 2014, informed our analyses. The main characteristics of the interviewees can be found in Additional file 1: Table S1.

These 27 surveys pooled self-reports from 334,606 eligible women and 2,048 reported having ever experienced VF symptoms (742 had missing information on the outcome (0.2 %)). The specific sample size used in the regression models varied, depending on the considered risk factors, from 332,889 for literacy to 102,928 for intimate partner sexual violence (before matching). Detailed information on the risk factors and number of women reporting VF symptoms, stratified by surveys, can be found in Table 1 and Table 2. Briefly, a little over a third of women were able to read (38.6 %), a quarter had completed post-primary education (26.8 %), 42.2 % had experienced FGM, 8.7 % had a height below 150 cm, 9.0 % of ever married women had experienced intimate partner sexual violence, 11.0 % of sexually active women had their first sexual intercourse before the age of 14, 3.8 % of primi/multiparous women had their first birth before the age of 14, and 18.3 % of women reported that obtaining permission to seek health care was a big problem for them.

### Risk factors for vaginal fistula

The sample size of the pooled datasets before and after matching are presented for each risk factors in a supplementary appendix (Additional file 1: Table S2). One-to-one matching was used for the risk factors that were most prevalent: being able to read, having a post-primary education, female genital mutilation, and degree of difficulty in obtaining permission to seek health care. For the other risk factors, the ratio was chosen as to minimize imbalances while retaining sufficient statistical power: one-to-two matching for intimate partner sexual violence, one-to-three for young age at first sexual intercourse, one-to-four for short stature, and one-to-eight for young age at first birth.

Results from the matched logistic regressions are presented in Table 3. Preliminary results from the Bayesian models for young age at 1^st^ birth and problem getting permission to seek healthcare suggest convergence issues with the country fixed effects. Since matched logistic regressions with and without country fixed effects for these two risk factors gave very similar results (data not shown), they were omitted from the Bayesian model.

**Table 3:** Matched logistic regression results for the different risk factors for vaginal fistula symptoms

Risk Factors	Bayesian matched logistic regressions adjusting for outcome misclassification
	**OR (95 % CrI)**
Being able to read	**0.87 (0.77-0.99)**
Post-primary education	0.90 (0.76-1.06)
Female genital mutilation	1.04 (0.82-1.30)
Short stature (<150 cm)	**1.31 (1.02-1.68)**
Intimate partner sexual violence^a^	**2.13 (1.60-2.86)**
Young age at 1^st^ intercourse (<14 years)^b^	**1.41 (1.16-1.71)**
Young age at 1^st^ birth (<14 years)^c^	**1.39 (1.04-1.82)**
Problem with permission to seek care	1.20 (0.99-1.47)

OR = Odds ratio; 95 % CI = 95 % Confidence Interval; 95 % CrI = 95 % Credible Intervals.

Statistically significant results at the alpha = 0.05 level are bolded.

The matched logistic regression models adjust for the following covariates: age, literacy status (except for ‘*Being able to read*’ and ‘*Post-primary education*’), location of residence (urban/rural), gravidity status (except for ‘*Young age at 1* ^*st*^ *birth*’), religion, and country (country fixed effects were omitted from the Bayesian regressions for ‘*Young age at 1* ^*st*^ *birth’* and ‘*Problem getting permission to seek healthcare’*).

^a^Among married and/or ever married women (or those in a union).

^b^Among sexually active women.

^c^Among primi/multiparous women.

Being able to read decreased the odds of VF by 13 % (95 % Credible Intervals (CrI): 1 % to 23 %). The impact of having completed some post-primary education also reduced the odds of VF by 10 % (95 % CrI: -6 % to 24 %) but the effect did not reach statistical significance. For these two determinants, it is likely that gravidity status lies on the causal pathway between literacy/education and occurrence of VF. If that is the case, the effect size measures reported above should be interpreted as the direct effect of literacy/education on VF (i.e., the effect not mediated through gravidity). By not controlling for gravidity status, we can calculate the total effect of literacy/education. The total effect of being literate is a 20 % reduction in the odds of VF (95 % CrI: 10 % to 30 %). For post-primary education, the total effect is a 21 % reduction in the odds of VF (95 % CrI: 7 % to 34 %).

FGM had little effect on the odds of VF, after adjusting for outcome misclassification. Women with a short stature had odds of VF that were 31 % (95 % CrI: 2 % to 68 %) higher than their taller counterparts. Among the sample of ever married women (or in a union), the odds of having had VF for those that experienced intimate partner sexual violence were 2.13 times higher than those that never had (95 % CrI: 1.60-2.86). This finding was confirmed in the subsample of 13 surveys that asked all women (never married and ever/currently married) if they had ever experienced sexual violence (from anyone) with an odds ratio of 2.22 (95 % CrI: 1.72-2.90). Among sexually active women, the odds of VF for those that had sexual intercourse before the age of 14 were 41 % (95 % CrI: 16 % to 71 %) higher than those that had a sexual debut at an older age. Expanding our sample by including women that have not begun their sexually active life had little impact on this effect size estimate (odds ratio (OR) = 1.38; 95 % CrI: 1.14-1.66). Both of these findings are in line with the one from age at first birth. Indeed, having had a first live birth before the age of 14 was associated with odds of VF that were 39 % higher (95 % CrI: 1.04-1.82) than those that had their first birth at older ages. Finally, having difficulty obtaining permission to seek health care was associated with increased odds of reporting VF symptoms but this effect did not reach statistical significance (OR = 1.20; 95 % CrI: 0.99-1.47).

## Discussion

### Main findings

Pooling data from up to 27 population-based surveys conducted in sub-Saharan Africa, we identified the following risk factors for VF: illiteracy, short stature, sexual violence, young age at first sexual intercourse, and young age at first birth. These results corroborate findings from previous studies on the importance of some individual-level risk factors for obstetric fistulas [12, 13, 15, 43]. Short stature, early sexual debut and young age at first birth are risk factors that are related, among other things (e.g., cultural practices, women’s status, access to family planning), to a woman’s anthropometry. Women that were young at first sex/birth, with immature pelvic bones, and women of short stature have increased incidence of cephalo-pelvic disproportion, which is a known risk for obstructed labor [44]. Illiteracy has been found to significantly increase the odds of VF and its effect went beyond that mediated by gravidity status. In contrast, we found no significant direct effect of post-primary education on VF occurrence. This could be explained by the fact that literacy was objectively measured whereas the quality of primary education in sub-Saharan Africa varies widely, even within the same geographical region [45]. Alternatively, it is possible that education beyond primary school has no impact on VF incidence, suggesting that fistula sufferers are the most disadvantaged of the disadvantaged. We did not evidence any relationship between FGM and VF. The DHS/MICS questionnaire, however, did not enable us to investigate if the most severe forms of FGM, such as infibulation and gishiri cutting, are risk factors for VF. Experience of intimate partner violence had a large effect on VF occurrence, as reported previously [31]. Taken together, these results suggest that empowerment and improvement of women’s status could play a key role in reducing the burden of VF in sub-Saharan Africa.

### Strengths and limitations

A number of strengths characterize this study. First, we have conducted what is believed to be the largest population-based analysis of risk factors for VF, pooling data from up to 23 countries (27 surveys) in sub-Saharan Africa. Second, we explicitly modeled uncertainty of self-reports of VF symptoms using a Bayesian Latent C lass model whereas the few other studies that have examined risk factors for VF using DHS/MICS surveys did not perform such adjustments. Finally, we used a doubly-robust method for inferences as a safeguard to bias of effect size estimates.

This study has some limitations. First, some of the estimates could be affected by reverse causality. This is mostly true for intimate partner sexual violence since the majority of other risk factors are likely to have preceded the occurrence of VF symptoms. Hence, it is possible that women with VF have a higher probability of being affected by intimate partner sexual violence as VF impacts their status within marriage and community [46], for example by creating financial stress and/or affecting women’s economic productivity. Living with fistula was found to interfere with sexual activity for 85.2 % of patients in a multi-country study [18]. Some physical and psychological consequences of VF persist after repair [47] and this could influence risk of sexual violence [18]. Second, we could not exclude from our sample fistulas that were not of obstetric origins as many surveys did not record the cause of VF symptoms. Since more than 90 % of VF in sub-Saharan Africa are from obstetric origins [10, 13], inclusion of VF from other causes should have little impact on our estimated effect size measures. Third, risk factors like intimate sexual violence, the degree of difficulty of obtaining permission to seek health care, and literacy were measured at the time of interview and we assumed these to be time-invariant. This assumption could be violated if these risk factors have changed since the women’s onset of VF symptoms. Finally, the cross-sectional nature of the surveys, coupled with potentially important within-country migration, prohibited us from examining the effect of a number of other risk factors such as access to health services, quality of health services, and coverage of maternal health interventions that may ultimately represent important barriers to the prevention and elimination of obstetric fistula in sub-Saharan Africa.

### Interpretation

VF embodies many of the challenges of the post-2015 agenda, and, more specifically, of the unfinished reproductive health agenda. Despite a decade of maternal health improvements [2, 3], poor access to and quality of health services is the norm in most low and middle income countries with antenatal and perinatal care being the least equitable interventions [48]. The third sustainable development goal (SDG) aims at reducing the maternal mortality ratio to less than 70 per 100,000 live birth and to ensure universal access to sexual and reproductive health-care services, including family planning [49]. The fifth goal also calls for achieving gender equality and women empowerment, with the elimination of all form of violence against women and girls and of harmful practices such as early and forced marriage [49]. Attaining these objectives could have important synergistic impacts to reduce incidence of obstetric fistula [50], but quality of care should be emphasized as poor vulnerable women are often attended by “*the most disenfranchised members of the health-care system*” [4]. The importance of family planning and antenatal care should also be stressed. Indeed, universal access to sexual and reproductive health is emphasized in both the third and fifth SDG. Alongside, access to comprehensive emergency obstetric care should be viewed as a form of prevention [51, 52]. Yet, our study highlighted that fistula prevention could be most effective if accompanied with enhanced efforts on education and women empowerment.

## Conclusions

Our study confirms a number of important individual-level risk factors for VF, while adding precision to the effect size estimates, using population-based data from a large number of countries in sub-Saharan Africa. Increasing literacy, delaying age at first sex/birth, and preventing sexual violence could contribute to the elimination of obstetric fistula if concomitant improvements in access to quality sexual and reproductive healthcare are ensured.

### Ethics approval and consent to participate

Informed consent was provided by all survey participants (or their guardian) before questionnaire administration. Further, all DHS survey protocols have been approved by the Internal Review Board of ICF International in Calverton (USA) and by the relevant country authorities for both DHS and MICS. Further information on the ethics approval can be found in the individual country reports published by DHS and MICS.

### Consent for publication

Not applicable.

### Availability of data and materials

Datasets containing individual-level records are in the public domain and can be obtained from The DHS Program (DHS surveys) and UNICEF (MICS surveys).

## Additional file

Additional file 1: Table S1. Characteristics of the study population, stratified by survey. **Table S2.** Number of surveys included in the analyses, sample size in the un-matched datasets, matching ratio, and matched sample size for the selected risk factors. (DOCX 22 kb)

## Abbreviations

CrI: credible intervals
DHS: Demographic and Health Survey
FGM: female genital mutilation
MICS: Multiple Indicators Cluster Survey
OR: odds ratio
SDG: Sustainable Development Goals
VF: vaginal fistula
