# Supplementary material for: Risk factors for vaginal fistula symptoms in Sub-Saharan Africa: a pooled analysis of national household survey data
Source: BMC Pregnancy Childbirth. 2016 Apr 21;16:82. doi: 10.1186/s12884-016-0871-6 (PMC4839076; doi:10.1186/s12884-016-0871-6)
Supplement: Additional file 1: Table S1. — Characteristics of the study population, stratified by survey. Table S2. Number of surveys included in the analyses, sample size in the un-matched datasets, matching ratio, and matched sample size for the selected risk factors. (DOCX 22 kb) [file 12884_2016_871_MOESM1_ESM.docx]

**Table S1**: Characteristics of the study population, stratified by survey.

| **Country, Survey, and Year** | **VF (%)** | **Mean age (years)** | **Urban (%)** | **Nulligravid (%)** | **Religion (%)** | | | |
| --- | --- | --- | --- | --- | --- | --- | --- | --- |
|  |  |  |  |  | **Christian** | **Muslim** | **Other** | **Missing** |
| Benin DHS 2011-12 | 0.77% | 29.0 | 42.6% | 23.7% | 55.6% | 23.6% | 20.8% | 0.0% |
| Burkina Faso DHS 2010 | 0.12% | 28.8 | 31.4% | 21.7% | 30.7% | 60.0% | 9.1% | 0.3% |
| Cameroon DHS 2011 | 0.36% | 28.0 | 50.4% | 26.3% | 74.9% | 19.4% | 5.4% | 0.3% |
| Chad MICS 2010 | 0.25% | 27.7 | 41.6% | 19.0% | 32.0% | 61.6% | 6.2% | 0.2% |
| Comoros DHS 2012 | 2.07% | 27.7 | 42.3% | 43.8% | 0.4% | 98.9% | 0.1% | 0.7% |
| Congo DHS 2011-12 | 0.25% | 29.2 | 32.8% | 16.0% | 93.1% | 0.9% | 5.9% | 0.0% |
| DRC DHS 2007 | 0.44% | 28.2 | 47.9% | 27.1% | 96.3% | 2.0% | 1.7% | 0.1% |
| Ethiopia DHS 2005 | 0.73% | 27.9 | 31.4% | 33.2% | 65.8% | 32.1% | 2.1% | 0.0% |
| Guinea DHS 2012 | 0.69% | 28.6 | 38.5% | 22.9% | 7.8% | 89.7% | 2.4% | 0.1% |
| Kenya DHS 2008-09 | 0.92% | 28.4 | 31.0% | 27.1% | 81.0% | 16.1% | 2.9% | 0.1% |
| Malawi DHS 2010 | 0.57% | 28.1 | 13.3% | 20.9% | 88.1% | 11.0% | 0.8% | 0.1% |
| Mali DHS 2006 | 0.12% | 28.4 | 35.3% | 19.6% | 3.4% | 91.4% | 4.7% | 0.6% |
| Mali DHS 2012-13 | 0.68% | 28.5 | 31.3% | 17.8% | 3.9% | 93.2% | 2.9% | 0.0% |
| Mauritania DHS* | 0.08% | 31.5 | 40.9% | 12.3% | 0.0% | 0.0% | 0.0% | 100.0% |
| Niger DHS 2006 | 0.22% | 28.5 | 35.0% | 21.1% | 0.7% | 98.3% | 0.6% | 0.4% |
| Niger DHS 2012 | 0.14% | 28.7 | 30.5% | 16.7% | 0.0% | 0.0% | 0.0% | 100.0% |
| Nigeria DHS 2008 | 0.43% | 28.6 | 31.4% | 27.5% | 51.4% | 46.3% | 1.8% | 0.5% |
| Rwanda DHS 2005† | 3.04% | 31.2 | 19.7% | 0.0% | 95.0% | 2.5% | 2.4% | 0.0% |
| Senegal DHS 2010-11 | 0.11% | 27.8 | 39.5% | 31.0% | 4.0% | 95.4% | 0.6% | 0.0% |
| Sierra Leone DHS 2013 | 0.68% | 28.3 | 40.6% | 24.8% | 22.1% | 77.3% | 0.3% | 0.2% |
| Swaziland MICS 2010§ | 1.72% | 31.7 | 38.9% | 0.0% | 0.0% | 0.0% | 0.0% | 100.0% |
| Tanzania DHS 2010 | 0.50% | 28.7 | 25.6% | 26.6% | 0.0% | 0.0% | 0.0% | 100.0% |
| Togo MICS 2010 | 0.36% | 29.4 | 35.0% | 25.9% | 46.9% | 16.5% | 36.5% | 0.1% |
| Togo DHS 2013-2014 | 0.98% | 29.4 | 37.9% | 25.2% | 55.7% | 19.1% | 25.2% | 0.0% |
| Uganda DHS 2006 | 2.37% | 28.2 | 17.0% | 23.5% | 86.7% | 11.4% | 1.9% | 0.1% |
| Uganda DHS 2011 | 1.91% | 27.9 | 29.5% | 25.1% | 85.6% | 13.5% | 0.9% | 0.0% |
| Zambia DHS 2013-2014 | 0.55% | 28.3 | 48.0% | 23.4% | 98.7% | 0.5% | 0.6% | 0.3% |

VF=vaginal fistula

Age and location of residence (urban/rural) did not have any missing values. 75 women had missing information on their gravidity status (<0.02%).

*Sample restricted to ever married women.

†Sample restricted to women who had a live birth in the previous five years.

§Sample restricted to ever pregnant women.

**Table S2**: Number of surveys included in the analyses, sample size in the un-matched datasets, matching ratio, and matched sample size for the selected risk factors.

| **Risk Factors** | **Number of surveys** | **Un matched** | | |  | **Matched datasets** | | | |  |
| --- | --- | --- | --- | --- | --- | --- | --- | --- | --- | --- |
|  |  | **Number VF** | **Number Exposed** | **Number Unexposed** |  | **Ratio (1:k)** | **Number VF** | **Number Exposed** | **Number Unexposed** |  |
| Being able to read | 27 | 2,043 | 128,583 | 204,306 |  | 1:1 | 1,735 | 128,583 | 128,583 |  |
| Post-primary education | 27 | 2,047 | 89,477 | 244,253 |  | 1:1 | 1,019 | 89,477 | 89,477 |  |
| Female genital mutilation | 19 | 1,357 | 102,458 | 140,216 |  | 1:1 | 973 | 102,458 | 102,458 |  |
| Short stature (<150 cm) | 23 | 1,140 | 16,565 | 172,998 |  | 1:4 | 533 | 16,565 | 66,260 |  |
| Intimate partner sexual violence* | 17 | 679 | 9,233 | 93,695 |  | 1:2 | 280 | 9,233 | 18,466 |  |
| Young age at 1^st^ sex (<14 years)† | 26 | 1,838 | 28,984 | 234,730 |  | 1:3 | 768 | 28,984 | 86,952 |  |
| Young age at 1^st^ birth (<14 years)§ | 26 | 1,814 | 9,278 | 234,893 |  | 1:8 | 583 | 9,278 | 74,224 |  |
| Problem permission seek healthcare | 21 | 1,745 | 49,916 | 222,788 |  | 1:1 | 629 | 49,916 | 49,916 |  |
| *Among married and/or ever married women.  †Among sexually active women.  §Nulliparous women are excluded from this analysis. | | | | | | | | | | |
